# Vertical distribution and tissue selection of *Helicoverpa zea* (Lepidoptera: Noctuidae) adult oviposition and neonates on soybean with an indeterminate or determinate growth habit

**DOI:** 10.1093/ee/nvae046

**Published:** 2024-05-22

**Authors:** Taynara Possebom, Dominic Reisig, Anders Huseth, Rachel Vann

**Affiliations:** Department of Entomology and Plant Pathology, North Carolina State University, Gardner Hall 2306, 100 Derieux Place, Raleigh, NC 27607, USA; Department of Entomology and Plant Pathology, North Carolina State University, Vernon G. James Research and Extension Center, 207 Research Station Road, Plymouth, NC 27962, USA; Department of Entomology and Plant Pathology, North Carolina State University, Campus Box 7630, Raleigh, NC 27695, USA; Department of Crop and Soil Sciences, North Carolina State University, Campus Box 7620, Raleigh, NC 27695, USA

**Keywords:** corn earworm, growth habit, behavior

## Abstract

*Helicoverpa zea* (Boddie) is a polyphagous pest that can cause serious damage to crops, including soybeans (*Glycine max* L.). In soybeans with both determinate and indeterminate growth habits, *H. zea* larvae are more commonly found on leaves compared to blooms, stems, and pods. Past research demonstrated that *H. zea* adults tend to oviposit near the upper part of the plant canopy in soybeans with a determinate growth habit. However, ovipositional selection on soybeans with an indeterminate growth habit is unknown. We hypothesized that *H. zea* would oviposit more uniformly throughout the canopy on indeterminate soybean growth habits due to more diffuse reproductive tissue. We planted field and greenhouse experiments with varieties sharing a similar relative maturity (2 maturity group [MG] 5.2 varieties and 2 MG 5.4/5.5 varieties) but with different growth habits. To test oviposition selection, adult *H. zea* females were allowed to oviposit under field and caged conditions. We counted the number of *H. zea* eggs and neonates on each plant tissue type from each of 3 equal parts in the plant canopy: upper, middle, and lower. In both experiments, eggs and neonates were most common on leaves at the top of the plant regardless of soybean growth habit. Consequently, ovipositional selection is likely independent of reproductive tissue availability, and patterns of oviposition through the canopy are similar in growth habits. An improved understanding of *H. zea* ecology in soybeans relative to indeterminate growth habits may improve recommendations for managing this significant pest of soybean.

## Introduction

*Helicoverpa zea* (Boddie) (Lepidoptera: Noctuidae), also known as corn earworm, was first described as a row crop pest from North America in 1850 (Mitter et al. 1993). In soybeans (*Glycine max* L.), larvae usually feed on reproductive tissues such as flowers and pods (Hardwick 1965, Eckel et al. 1992a, Capinera 2000, Murúa et al. 2021). *Helicoverpa zea* feeding on soybean reproductive structures can lead to yield loss if the plants cannot compensate for feeding damage (Eckel et al. 1992b). From 2018 to 2021, *H. zea* was responsible for an estimated 51% to 56% of soybean insect management costs and losses in North Carolina, totaling an estimated $117–189 million in annual economic loss for farmers (Musser et al. 2019, 2020, 2021, 2022).

Soybean varieties vary based on the timing of when they begin reproductive development and morphological growth habit (Stowe and Vann 2022) and are classified by maturity group (MG) depending on when they begin reproductive development. Soybean MG categories vary from MG 000 to MG 10 and are based on varietal adaptation to certain latitudes (Boerma and Specht 2004). Soybean varieties can also be classified as determinate or indeterminate based on their morphological growth habits. Determinate varieties terminate vegetative growth on the main stem once the reproductive stages begin. Indeterminate varieties flower for a longer duration before terminating their vegetative growth on the main stem and tend to have longer stems and a prolonged duration of reproductive tissue availability (Fehr and Caviness 1977, Fehr et al. 1977, Hartung et al. 1981). Historically, soybean varieties with determinate growth habits and with an MG 5 or greater were the most commonly planted in North Carolina (Stowe and Vann 2022). However, indeterminate soybean varieties with an MG 5 or earlier are increasingly being planted in North Carolina because they tend to yield better under limited stress (Vann et al. 2021).

Artificial infestation studies demonstrated that *H. zea* tends to oviposit during the flowering reproductive stage of corn, tobacco (*Nicotiana tabacum*), and cotton (Parsons 1939, Hardwick 1965, Johnson et al. 1975). Leaves are the preferred *H. zea* oviposition site in soybeans with a determinate growth habit, snap beans (*Phaseolus vulgaris* L.), strawberries (*Fragaria* × *ananassa* Duch.), tomatoes (*Lycopersicon esculentum* Mill.), and cotton (*Gossypium hirsutum* L.) (Hillhouse and Pitre 1976, Alvarado-Rodriguez et al. 1982, Farrar and Bradley 1985, McLeod 1988, Wiesenborn and Trumble 1989, Eckel et al. 1992c, Braswell 2019b). In contrast, in corn (*Zea mays* L.) sweet corn (*Zea mays* convar. saccharata var. rugosa), *H. zea* females tend to lay individual eggs on leaves, trichomes, and silks (Quaintance and Bishopp 1905, Nishida and Napompeth 1974, Herbert et al. 2009).

In soybean varieties with a determinate growth habit, *H. zea* females tend to lay more eggs from the R1 to R3 stages (beginning flowering to beginning pod) compared to the R3 to R6 stages (beginning pod to full seed) (Johnson et al. 1975, Fehr and Caviness 1977). In a study comparing both growth habits, the timing of oviposition was inferred based on place during the bloom period (Reisig et al. 2021). However, the timing of oviposition was determined solely through indirect evidence, relying on *H. zea* larval measurements and counts as plant growth progressed. Indeterminate soybeans, *H. zea* preferentially oviposits in the top of the canopy, primarily on the upper main stem trifoliate, and in the lower part of the canopy *H. zea* primarily oviposits on the lower main stem trifoliate, during reproductive soybean stages (Terry et al. 1987a). After hatching, larvae produce silk and move down through the canopy using a spin-down behavior toward the soil, perhaps to search for other tissue available to feed on (Terry et al. 1989). This spin-down behavior shortly after hatching may explain why *H. zea* larvae are equally distributed throughout the soybean canopy (Reisig et al. 2021).

No study has directly compared ovipositional selection for *H. zea* adults between growth habits. One hypothesis is that moths tend to lay eggs in indeterminate varieties as they flower over an extended period. This hypothesis is supported by the fact that plant nectar can be an important nutritional resource for *Heliothis* spp. moths (Adjei-Maafo and Wilson 1983a, 1983b). In addition, indeterminate varieties might offer multiple tissue options for the offspring of *H. zea* to feed on compared to determinate varieties. For example, indeterminate varieties have both flowers and pods in the lower canopy (University of Wisconsin 2023). In contrast, determinate varieties have much more discrete periods of flowering followed by pod development. Feeding experiments using detached tissues have shown that 2nd instar *H. zea* larvae tend to feed on newly emerging soybean trifoliate and early developing pods, while 4th instar larvae do not exhibit any preference among tissue types (Suits et al. 2017). Hence, *H. zea* might develop an ovipositional behavior on indeterminate growth habits due to a wide variety of soybean tissue types for optimal offspring nutrition and growth.

We developed a complementary controlled greenhouse cage study and field infestation study to determine oviposition distribution on indeterminate soybeans. We had 2 research goals: (1) evaluating the vertical distribution of *H. zea* eggs and neonates on soybeans and (2) evaluating the location of *H. zea* eggs and neonates on various soybean tissues. Because reproductive tissue availability varies between growth habits, we hypothesized that (1) *H. zea* vertical distribution would differ between soybean growth habits and (2) *H. zea* would choose to oviposit across different tissue types between growth habits. In both studies, we tested soybean varieties with determinate and indeterminate growth habits that shared similar relative maturity (2 MG 5.2 varieties and 2 MG 5.4/5.5 varieties). We counted the number of *H. zea* eggs and neonates in 3 different vertical distribution categories (upper, middle, and bottom of the soybean plant) and across 4 tissue categories (adaxial leaves, abaxial leaves, stem, and reproductive tissues). Together, these studies help define oviposition selection differences between determinate and indeterminate growth habits that may improve the accuracy of *H. zea* scouting and threshold decisions.

## Materials and Methods

### Controlled Greenhouse Cage Study

We used a completely random design for our greenhouse experiments in 2021. We used varieties that shared a similar MG, 2 MG 5.2 varieties, and 2 MG 5.4/5.5 varieties but had different growth habits—determinate (5220R2X/SR and AG55XF0, Bayer Crop Science St. Louis, MO, USA) and indeterminate (AG52XF0 XF and AG54XF0 XF, Bayer Crop Science). All varieties were tolerant to dicamba, glyphosate, and glufosinate.

Prior to planting, we used samples sent to the North Carolina Department of Agriculture for chemical analyses to calculate lime and fertilizer amendment requirements recommended by the North Department of Agriculture (North Carolina Department of Agriculture 2009). We planted 2 soybean seeds of a single variety in a single clay pot (15.24 cm width × 12.70 cm height and 1957 cubic cm volume) but thinned them to only 1 plant prior to flowering. We performed fertilization during the soybean stages V7–V8 (soybean plants had 7–8 fully developed trifoliolate leaf nodes). We applied nitrogen in 60 mg of urea/L (Cz Garden, MI, USA) in 10 mg/pot every 15 days starting during the soybean stage V7–V8. Once the plants were almost blooming (V8-R1), we randomly placed 2 pots of each of the 4 varieties in each cage (121 cm width and height × 243 cm length, 4 cages total, each containing 2 pots with 2 plants, each randomly arranged of each of the 4 soybean varieties, a total of 16 plants per cage, soybean plants were not touching each other inside of the cage).

In the laboratory, we reared *H. zea* since 2019 (about 6 generations/year) at 26 ± 5 °C and 65% ± 5% RH, with a photoperiod of 12:12 (L:D) cycle, and we fed the larvae on an artificial diet (*H. zea* diet, Southland Products, Lake Village, AR, USA). Once the larvae were pupated, we determined their sex and then waited approximately 2 wk until the pupae neared eclosion. Two days before eclosion, we placed 1 female and 1 male pupa into open Petri dishes and added a single Petri dish to the top of the soil of each clay pot caged at the greenhouse (total density of 8 males and 8 females per cage, 16 total). Before adult eclosion, the soybean varieties approached full flowering (R2). Within a day or 2, moths emerged. We checked emergence after 24 h and 48 h to make sure we had between 14 and 16 individuals per cage; emergence varied from 87.5% to 100%, and we repeated this experiment 4 times. Emergence dates were 9–10 June, 27–28 July, 19–20 August, 25–26 August 2021. Three days following adult emergence, we counted the total number of eggs oviposited on plants and neonates (12 June, 30 July, 22 August, and 28 August 2021). We used the cage as the level of replication, with 2 subreplicates (example: 2 plants of 5220R2X/SR per pot, 2 plants of AG55XF0 per pot, 2 plants of AG52XF0 XF, and 2 plants of AG54XF0 per pot) in each cage (*n* = 8 plants/cage), and we had 4 replicates during each run of the experiment.

### Data Collection—Controlled Greenhouse Cage Study

We destructively sampled each soybean plant in 3 equal parts—upper, middle, and lower depending on the number of nodes/plants. For example, in a plant with 12 nodes, the lower part included node number 4 and below, the middle part included node number 8 to node number 5, and the top 4 nodes were considered the upper part of the plant. If there was an odd number of nodes, for example, a plant with 13 nodes, the lower part included node number 4 and a half of the next node or 4.3, and the middle part included node number 5 and half of the previous node to node number 8, and the top 4 nodes were considered the upper part of the plant including half of the previous node as well. Basically, we divided the plant into equal parts if the number of nodes was not even number. We collected the data once for each of the 4 experiments (experiment-sampling dates: 12 June, 30 July, 22 August, and 28 August 2021) from 8:00 to 12:00. In a sampling event, we recorded the total number of *H. zea* eggs and neonates in each of 4 tissue categories—adaxial leaves, abaxial leaves, stem, and reproductive tissues—for each of the 3 vertical plant categories (a small number of eggs were in the cage walls/mesh, but we did not record it). Note that we did not classify expanded and newly emerged trifoliate leaves into separate categories but subsumed them into the categories of adaxial and abaxial leaves. Petioles, main stems, and lateral stems were subsumed into a single category of stems. Finally, we pooled flowers and pods in a single category of reproductive tissues.

### Field Study

We used a randomized complete block design for our field experiments at 4 locations in North Carolina in 2021 (4 blocks × 4 soybean varieties, 16 plots). We planted with a commercial research planter during mid-June at the Central Crops Research Station in Clayton (Wake County), Tidewater Research Station in Plymouth (Washington County), Upper Coastal Plains Research Station in Rocky Mount (Edgecombe County), and the Roper farm area (Washington County). We used the same varieties as our cage studies. We used a consistent experimental design among locations, with each plot measuring 12.19 m in length and 8 rows in width, with 0.91 m spacing between rows; these fields were no-till.

We maintained fields according to the guidelines recommended by the North Carolina Cooperative Extension Service (Stowe et al. 2018). Before planting, we sprayed the preemergence herbicide S-metolachlor (2.33 L/ha of Prefix, Syngenta, DE, USA). One month later, we sprayed the postemergent herbicides glyphosate (2.19 L/ha of Roundup, Bayer Crop Science) and cloransulam-methyl (0.21 L/ha of FirstRate, Corteva Agriscience, IN, USA). We did not apply any insecticides in those fields, with one exception. In Wake County, *Megacopta cribraria* (Fabricius) (kudzu bug) reached the treatment threshold during the soybean stages V7–V8, and we sprayed the insecticide bifenthrin (1.96 L/ha of Quali-Pro Bifenthrin, Makhteshim Agan of North America, NC, USA) to avoid potential confounding effects from *M. cribraria*. We relied on natural infestations of *H. zea* in the experiments.

### Data Collection—Field Study

In the natural infestation studies, we spot-checked our fields weekly for the first appearance of *H. zea* eggs and larvae. Once eggs appeared, we randomly selected 10 plants from the 2 middle rows (*n* = 10 plants/plot) and divided each soybean plant into 3 equal parts—upper, middle, and lower depending on the number of nodes/plant, similar to the controlled greenhouse cage experiments. We sampled for eggs and neonates during the beginning of soybean reproductive stages (R1 or R2) at only one time in each location, from 8:00 to 15:00. The criterion used to determine if the larvae were a neonate was their size (1 to 3 cm) (Hardwick 1965). Additionally, we collected a subset of egg samples (10 eggs/plot in each location, or 640 eggs) from the field that we reared for later species confirmation. All larvae that we reared were *H. zea*. Similar to cage experiments, we recorded the total number of *H. zea* eggs and neonates in each of 4 tissue categories—adaxial leaves, abaxial leaves, stem, and reproductive tissues—for each of the 3 vertical plant categories.

### Statistical Analysis

We calculated the average number of eggs and the total number of neonates per plant within a variety in each cage (*n* = 2 plants) or each plot (*n* = 10 plants) for each category by vertical distribution (upper, middle, bottom), and tissue type (adaxial leaves, abaxial leaves, stem, reproductive tissue). We conducted all our analyses with RStudio version 1.2.5042 (RStudio Core Team 2020). We analyzed the data using the generalized linear mixed model for zero-inflated combined with Poisson regression using the *glmmTMB* package (Brooks et al. 2017, 2023) and ANOVA with the *car* package (Fox and Weisberg 2019). We performed 4 individual analyses for each of our categories. Our dependent variables included the total number of eggs or neonates (mean number of eggs for our cage study, mean number of neonates for our cage study, mean number of eggs for our natural infestation study, and mean number of neonates for our natural infestation study). Our independent variables included soybean growth habit (determinate and indeterminate), vertical distribution (upper, middle, and lower), and tissue type (adaxial leaves, abaxial leaves, stem, reproductive tissue), as well as biologically relevant 2-way interactions (soybean growth habit × vertical distribution; soybean growth habit × tissue type; and vertical distribution × tissue type). We included experimental replication nested within the location as a random effect for the natural infestation study. We included replication nested within experimental replicate (4 experiments) as a random effect for the cage study. We performed the Tukey’s HSD test at a significance level of 5% (*α* = 0.05) for mean separations, using the *multcomp* and *glht* packages (Hothorn et al. 2008, Lee and Kyu Lee 2018).

## Results

### Controlled Greenhouse Cage Study

#### Helicoverpa zea egg distribution

We did not observe a significant interaction between the main effects of vertical distribution (upper, middle, and lower) and tissue type (adaxial leaves, abaxial leaves, stem, and reproductive tissue) for the mean total number of *H. zea* eggs (Table 1). However, the main effects of vertical distribution and tissue type differed for the mean total number of *H. zea* eggs (Table 1). We found most eggs on the upper part of the canopy (3.28 average number of *H. zea* eggs per plant ± 0.76 SE), compared to the middle (0.57 ± 0.66) and lower part of the canopy (0.20 ± 0.38). We found most eggs on abaxial (3.16 ± 0.57) and adaxial (2.59 ± 0.63) leaves compared to all other tissues (stem 0.65 ± 0.64, reproductive tissues 0.60 ± 0.43).

**Table 1.** Analysis of variance results for *Helicoverpa zea* eggs and neonates in controlled greenhouse cage study and in field study with growth habit (determinate and indeterminate), vertical distribution (upper, middle, and lower), tissue type (abaxial leaflet, adaxial leaflet, stem, and reproductive tissues which include flowers and young pods) and the 2-way interactions between growth habit and vertical distribution, growth habit and tissue type, vertical distribution, and tissue type

Dependent variable	Independent variables	*Df*	*x* ^2^	*P*-value
Total number of eggs in controlled greenhouse cage experiments	Growth habit (Gh)	1	0.48	0.481
	Vertical distribution (Vd)	2	273.83	<0.001***
	Tissue type (Tt)	3	161.70	<0.001***
	Gh * Vd	2	4.34	0.112
	Gh * Tt	3	7.89	0.044*
	Vd * Tt	6	7.95	0.240
Total number of neonates in controlled greenhouse cage experiments	Growth habit (Gh)	1	1.44	0.223
	Vertical distribution (Vd)	2	374.18	<0.001***
	Tissue type (Tt)	3	210.19	<0.001***
	Gh * Vd	2	2.83	0.242
	Gh * Tt	3	8.46	0.037*
	Vd * Tt	6	49.45	<0.001***
Total number of eggs in natural field experiments	Growth habit (Gh)	1	0.08	0.765
	Vertical distribution (Vd)	2	106.03	<0.001***
	Tissue type (Tt)	3	117.33	<0.001***
	Gh * Vd	2	0.34	0.840
	Gh * Tt	3	11.01	0.010*
	Vd * Tt	6	7.99	0.233
Total number of neonates in natural field experiments	Growth habit (Gh)	1	0.90	0.344
	Vertical distribution (Vd)	2	138.05	<0.001***
	Tissue type (Tt)	3	131.91	<0.001***
	Gh * Vd	2	3.50	0.173
	Gh * Tt	3	11.26	0.010*
	Vd * Tt	6	36.60	<0.001***

Significance levels: *P ≤ 0.05, ***P ≤ 0.001.

**Fig. 1.**
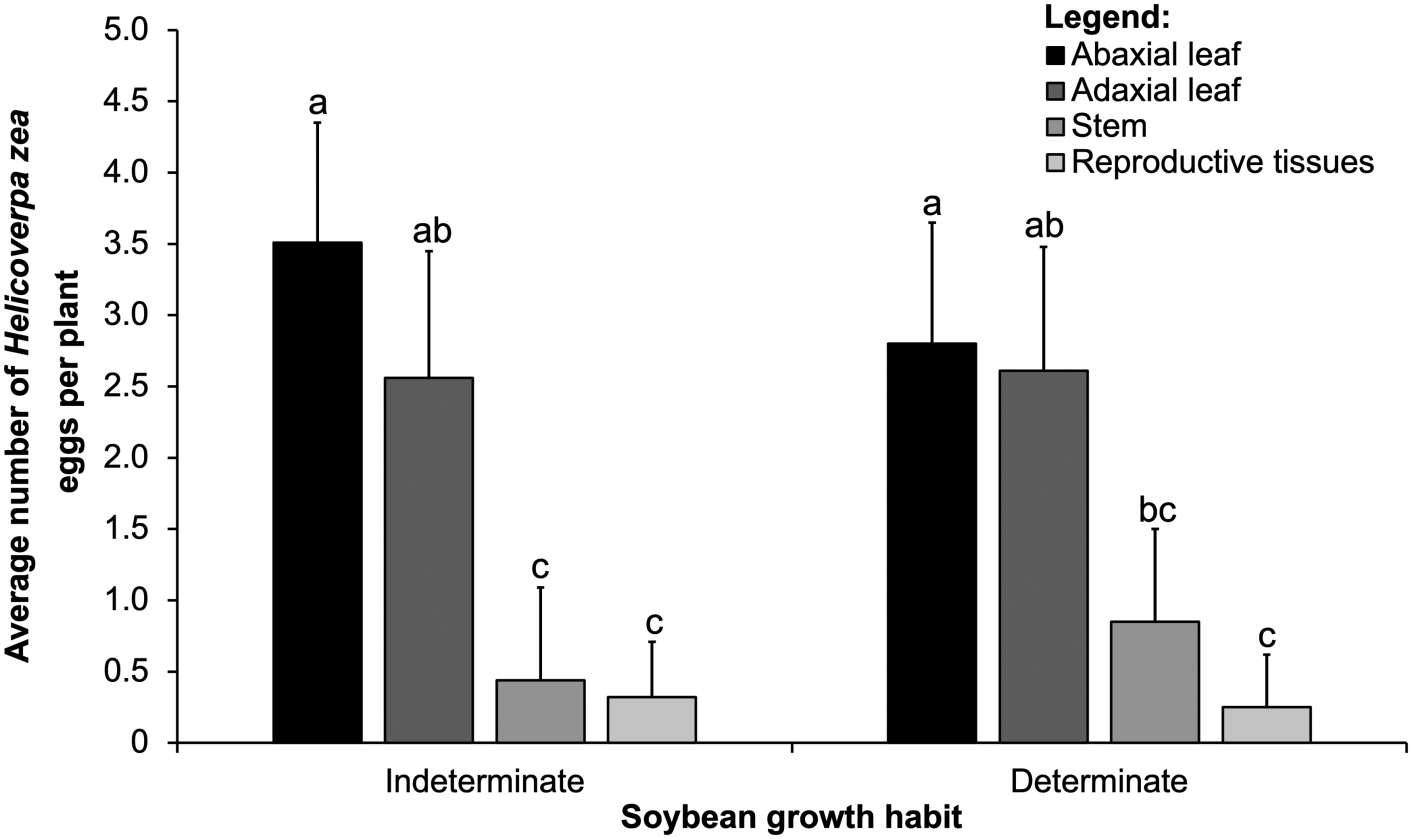
Interaction between soybean growth habit (*x*-axis) and tissue type (abaxial leaflet, adaxial leaflet stem, and reproductive tissues that include flowers and young pods) in controlled greenhouse cage study. Means (± SE, *y*-axis) of *Helicoverpa zea* eggs within each tissue for each vertical distribution followed by the same letter are not significantly different using the Tukey’s HSD test, *P* < 0.05.

We observed a significant interaction between the main effects of soybean growth habit (indeterminate and determinate) and tissue type for the mean total number of *H. zea* eggs (Table 1). We found that most eggs were on abaxial and adaxial leaves, compared to stem and reproductive tissues, in both growth habits (Fig. 1). The interaction was significant because we found more eggs were laid on the adaxial leaves compared to stems in indeterminate growth habit, but we found similar numbers of eggs on adaxial leaves and stem tissues in determinate growth habit (Fig. 1). We did not observe a significant interaction between the main effects of soybean growth habit and vertical distribution for the mean total number of *H. zea* eggs (Table 1), and egg numbers did not differ significantly for the main effect of soybean growth habit (Table 1).

#### Helicoverpa zea neonates distribution

We observe a significant interaction between the main effects of vertical distribution and tissue type for the mean total number of *H. zea* neonates (Table 1). We found most neonates on the upper part of the canopy (23.54 average number of *H. zea* neonates per plant ± 0.90 SE), compared to the middle (7.23 ± 0.78) and lower part of the canopy (2.07 ± 0.74). Also, we found most neonates on the adaxial (5.01 ± 0.63) and abaxial (3.44 ± 0.57) leaves compared to all other tissues (stem 1.35 ± 0.64, reproductive tissues 1.04 ± 0.45).

**Fig. 2.**
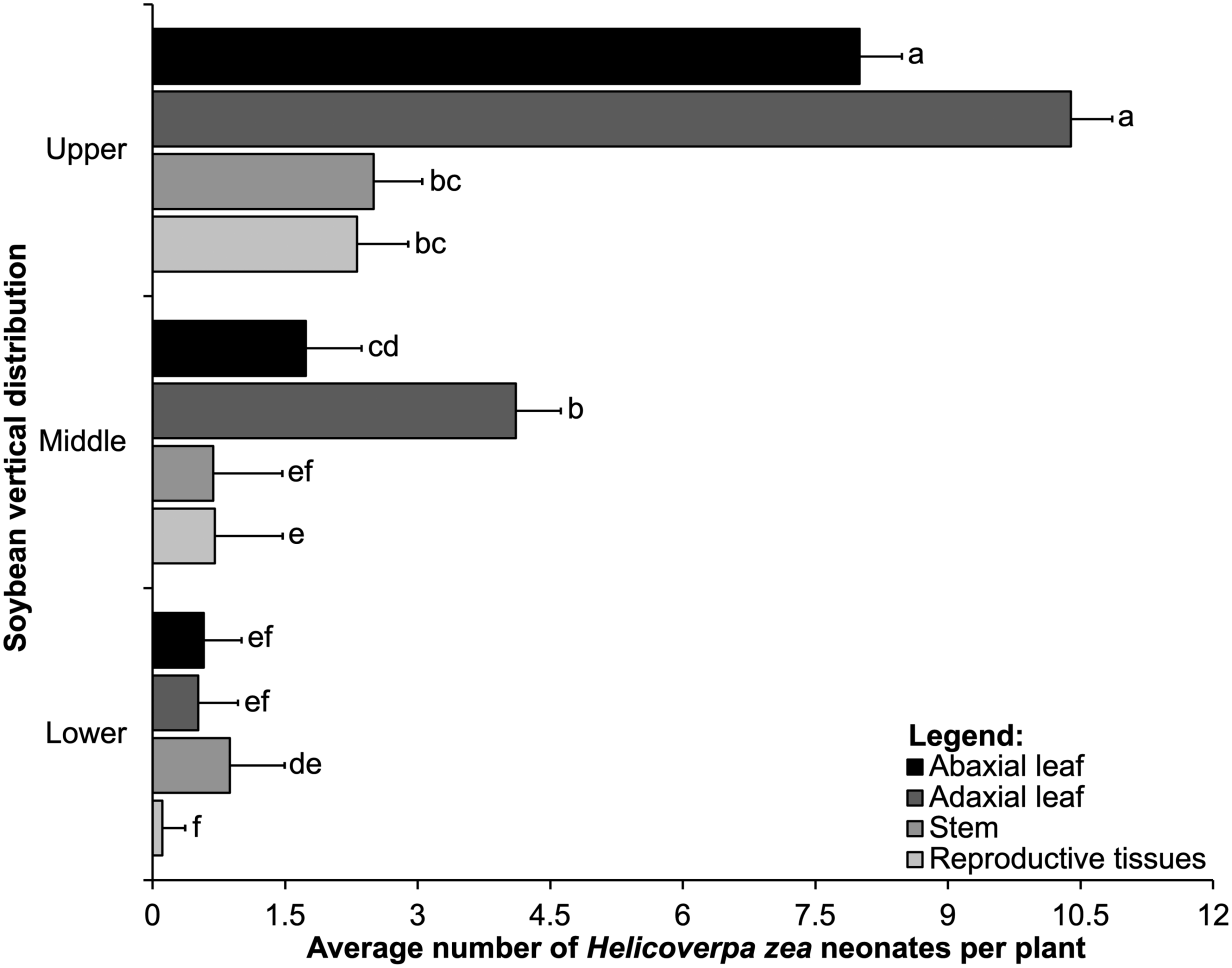
Interaction between soybean vertical distribution (*y*-axis) and tissue type (abaxial leaflet, adaxial leaflet, stem, and reproductive tissues that include flowers and young pods) in controlled greenhouse cage study. Means (± SE, *x*-axis) of *Helicoverpa zea* neonates within each tissue for each vertical distribution followed by the same letter are not significantly different using the Tukey’s HSD test, *P* < 0.05. This result combined both growth habits.

We observed a significant interaction between the main effects of vertical distribution and tissue type for the mean total number of *H. zea* neonates (Table 1). The interaction was significant because we found more neonates on stems than reproductive tissues in the lower part of the canopy (Fig. 2).

**Fig. 3.**
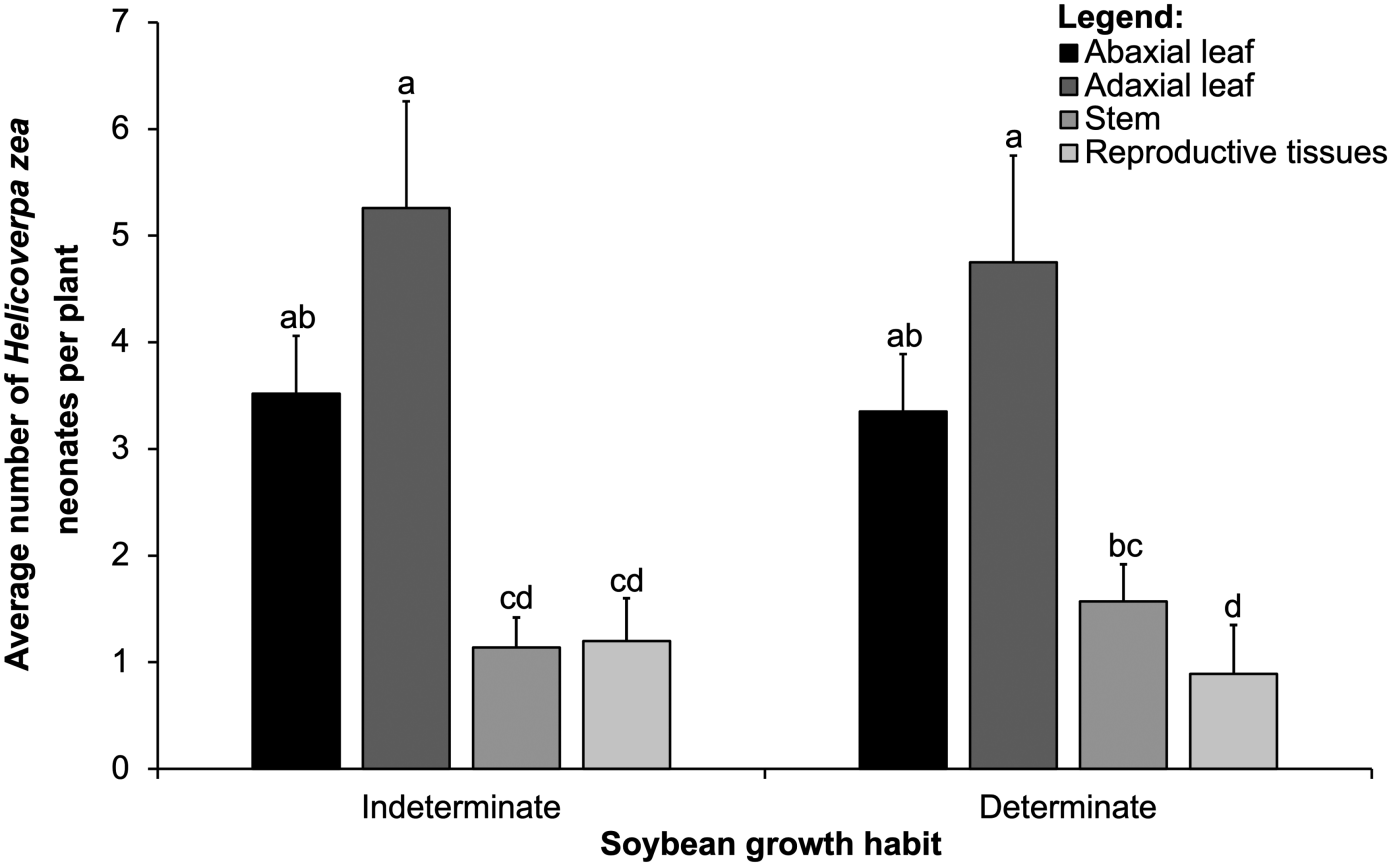
Interaction between soybean growth habit (*x*-axis) and tissue type (abaxial leaflet, adaxial leaflet stem, and reproductive tissues that include flowers and young pods) in controlled greenhouse cage study. Means (± SE, *y*-axis) of *Helicoverpa zea* neonates within each tissue for each vertical distribution followed by the same letter are not significantly different using the Tukey’s HSD test, *P* < 0.05.

We did not observe a significant interaction between the main effects of soybean growth habit and vertical distribution for the mean total number of *H. zea* neonates (Table 1). Neonate numbers did not differ significantly in terms of the main effect of soybean growth habit (Table 1). We observed a significant interaction between the main effects of soybean growth habit and tissue type for the mean total number of *H. zea* neonates (Table 1). We found most neonates on adaxial and abaxial leaves, compared to stem and reproductive tissues (Fig. 3). We found a significant interaction because more neonates were on stems compared to reproductive tissues in the determinate growth habit but not in the indeterminate growth habit (Fig. 3).

### Field Study

#### Helicoverpa zea egg distribution

We did not observe a significant interaction between the main effects of soybean vertical distribution and tissue type for the mean total number of *H. zea* eggs (Table 1). However, the total number of *H. zea* eggs differed significantly in terms of the main effects of vertical distribution and tissue type (Table 1). We found most eggs on the upper part of the canopy (0.90 average number of *H. zea* eggs per plant ± 0.34 SE), compared to the middle (0.45 ± 0.25) and lower part of the canopy (0.10 ± 0.10). Also, we found most eggs on the adaxial (1.33 ± 0.30) and abaxial (0.86 ± 0.23) leaves compared to all other tissues (stem 0.16 ± 0.11, reproductive tissues 0.03 ± 0.01).

**Fig. 4.**
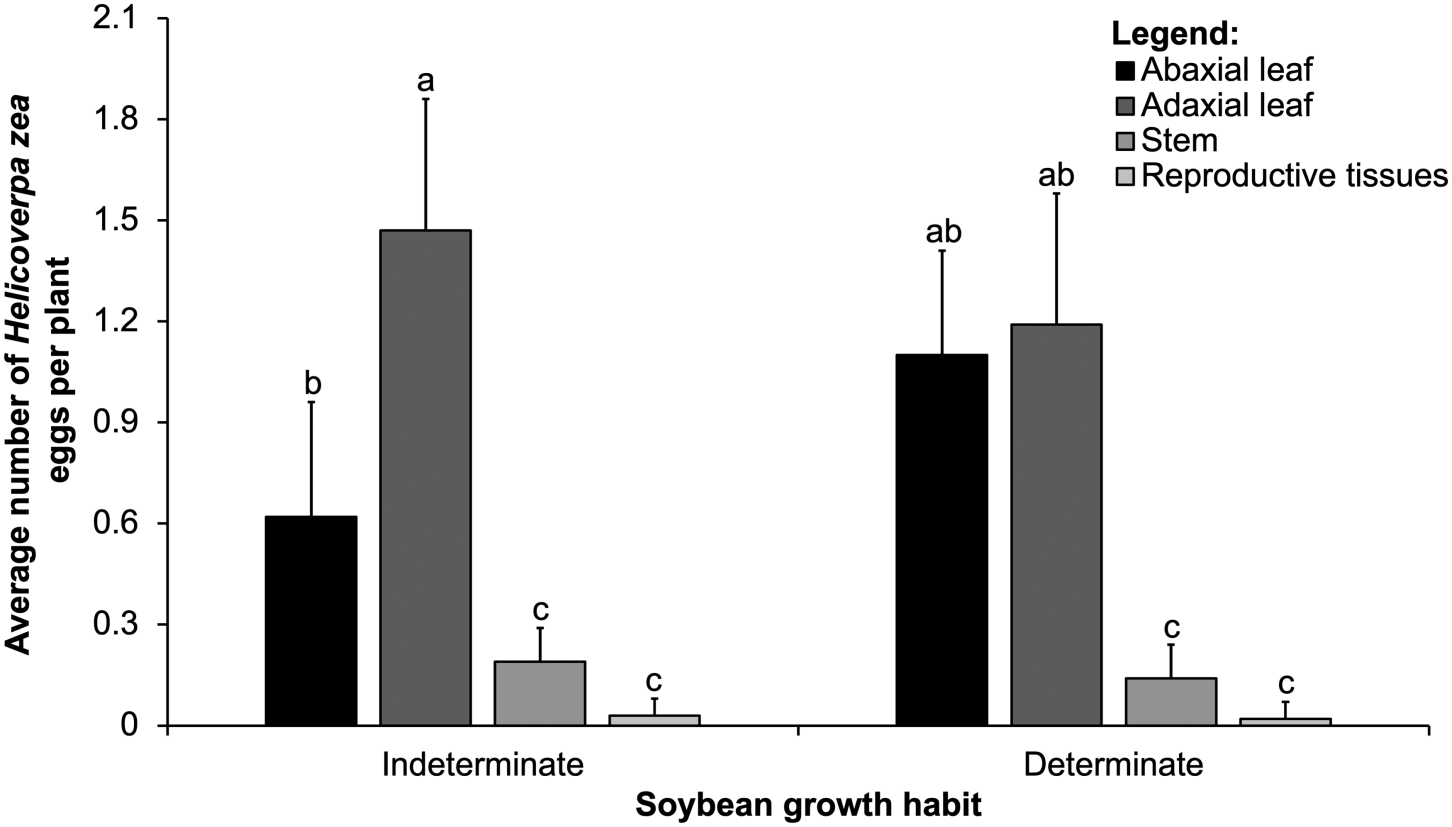
Interaction between soybean growth habit (*x*-axis) and tissue type (abaxial leaflet, adaxial leaflet, stem, and reproductive tissues that include flowers and young pods) in field study. Means (± SE, *y*-axis) of *Helicoverpa zea* eggs within each tissue for each vertical distribution followed by the same letter are not significantly different using the Tukey’s HSD test, *P* < 0.05.

We observed a significant interaction between the main effects of soybean growth habit and tissue type for the mean total number of *H. zea* eggs (Table 1). However, egg numbers did not differ significantly in terms of the main effects of soybean growth habit. We found most eggs laid on abaxial and adaxial leaves, compared to stem and reproductive tissues, in both growth habits (Fig. 4). We found a significant interaction because fewer eggs were found on the abaxial leaves compared to the adaxial leaves in soybeans with an indeterminate growth habit (Fig. 4).

We did not observe a significant interaction between the main effects of soybean growth habit and vertical distribution for the mean total number of *H. zea* eggs (Table 1), and egg numbers did not differ significantly for the main effect of soybean growth habit (Table 1).

#### Helicoverpa zea neonates’ distribution

**Fig. 5.**
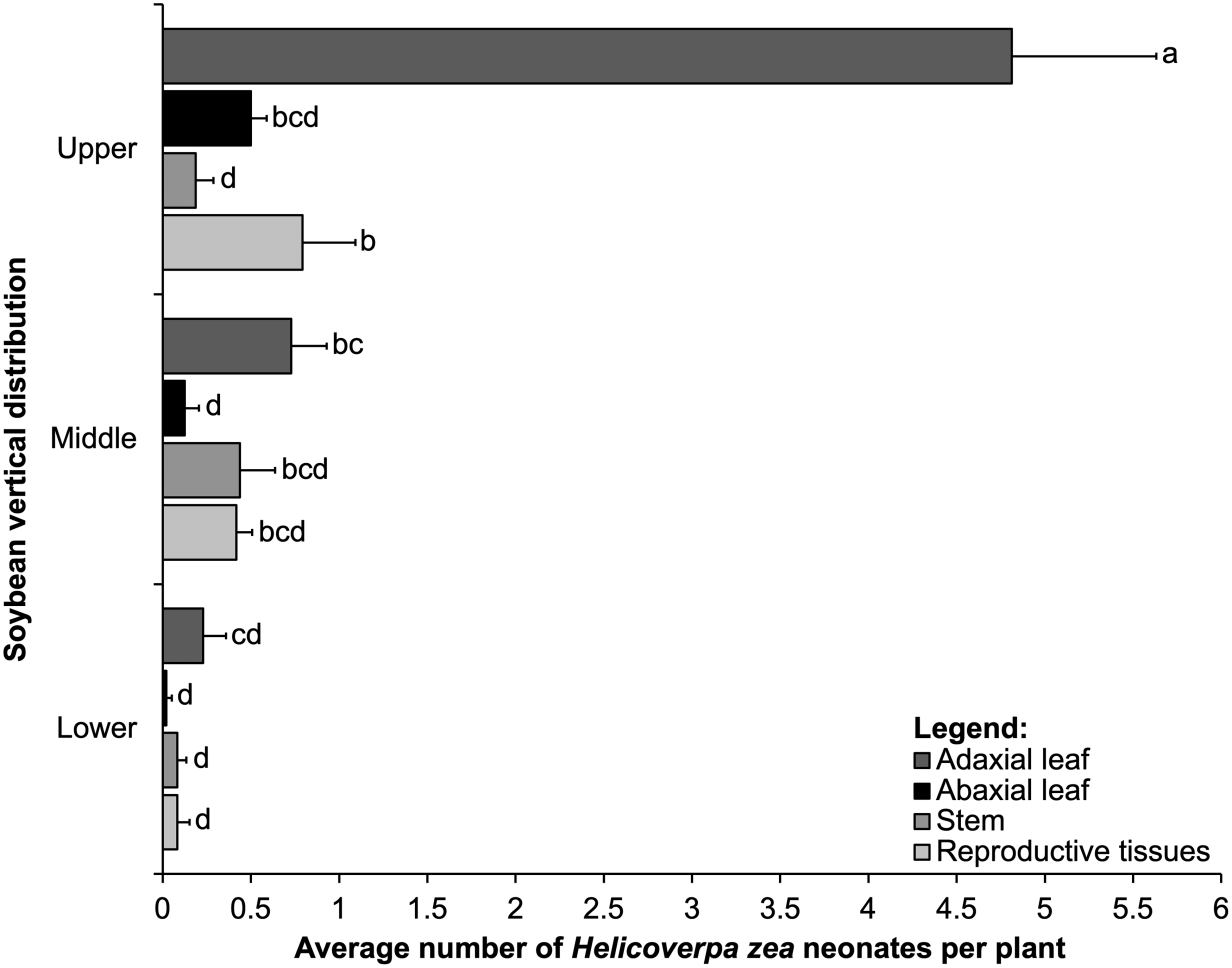
Interaction between vertical distribution (*y*-axis) of *Helicoverpa zea* neonates and tissue type (abaxial leaflet, adaxial leaflet, stem, and reproductive tissues that include flowers and young pods) in the field study. Means (± SE, *x*-axis) of *H. zea* neonates within each vertical distribution for each tissue followed by the same letter are not significantly different using the Tukey’s HSD test, *P* < 0.05. This result combined both growth habits.

Additionally, we observed a significant interaction between the main effects of vertical distribution and tissue type for the mean total number of *H. zea* neonates (Table 1). We found most neonates on the upper part of the canopy on the adaxial leaves compared to all other vertical distributions and tissues (Fig. 5). We found a significant interaction because fewer neonates were on adaxial leaves in the lower part of the canopy compared to adaxial leaves in the upper part of the canopy; also, we found more neonates in reproductive tissues in the upper part of the canopy compared to abaxial leaves (Fig. 5). The total number of *H. zea* neonates differs significantly for the main effects of vertical distribution and tissue type (Table 1). We found most neonates on the upper part of the canopy (6.29 average number of *H. zea* neonates per plant ± 0.55 SE), compared to the middle (1.71 ± 0.35) and lower part of the canopy (0.42 ± 0.10 SE). Most neonates were on the adaxial leaves (1.93 ± 0.53) compared to all other tissues (abaxial leaves 0.21 ± 0.10, stem 0.24 ± 0.11, reproductive tissue 0.43 ± 0.20).

**Fig. 6.**
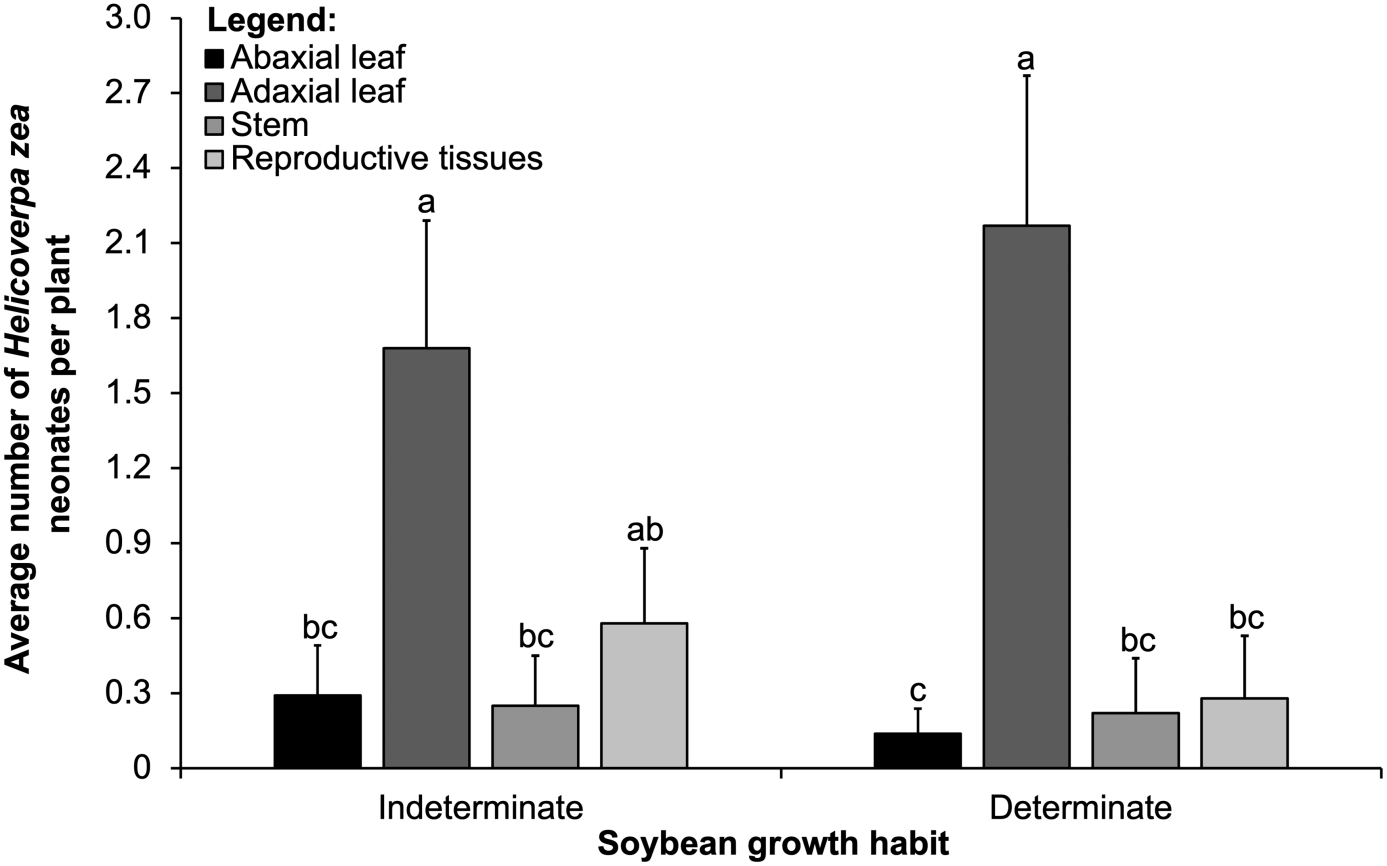
Interaction between soybean growth habit (*x*-axis) and tissue type (abaxial leaflet, adaxial leaflet, stem, and reproductive tissues that include flowers and young pods) in the field study. Means (± SE, *y*-axis) of *Helicoverpa zea* neonates within each tissue for each vertical distribution followed by the same letter are not significantly different using the Tukey’s HSD test, *P* < 0.05. This result combined both growth habits.

We observed a significant interaction between the main effects of soybean growth habit and tissue type for the mean total number of *H. zea* neonates (Table 1). However, neonate numbers did not differ significantly in terms of the main effects of soybean growth habit. We found most neonates on adaxial leaves compared to all other tissues (Fig. 6). We found a significant interaction because neonate numbers were statistically similar on adaxial leaves and reproductive tissues in the indeterminate growth habit but not in the determinate growth habit (Fig. 6). We did not observe a significant interaction between the main effects of soybean growth habit and vertical distribution for the mean total number of *H. zea* neonates (Table 1), and neonate numbers did not differ significantly for the main effect of soybean growth habit (Table 1).

## Discussion

We found that egg and neonate location, both vertically within the plant and across tissue type, was consistent between growth habits. Across all relative maturities (5.2, 5.4, and 5.5) and between both growth habits, females tended to lay eggs in the upper part of the canopy and on leaves. Most neonates were also in the upper part of the canopy and on leaves. Since we sampled these very soon after hatching, we assumed that they had not yet spun down the canopy to feed on reproductive tissues and leaves (Terry et al. 1989). If this is true, and if we had extended our sampling period, we expected the second and third instar of *H. zea* larvae to be evenly vertically distributed within the soybean plant but more often encountered on soybean leaves, which aligns with our study (Reisig et al. 2020, 2021). Finally, these effects were remarkably consistent across wild (field study) and laboratory-reared (cage studies) *H. zea* populations.

Most of the interactions for tissue selection were due to differences in distribution in the middle and lower part of the canopy, where we found very few eggs and neonates. Therefore, while many of the interactions we tested were statistically significant, most of them were not as strong as the results in the main effects, particularly the vertical distribution of eggs and neonates, which has implications for scouting. Our research yielded the following regarding egg distribution: In the case of the cage study, 86% of eggs were deposited on leaves, with 81% of them located in the upper part of the canopy, 14% in the middle, and 5% in the lower canopy. In the field study, 89% of eggs were laid on leaves, and 62% of these were found in the upper canopy. It is worth noting that similar to the cage study findings, only a small percentage of eggs were discovered in the middle (31%) and lower (7%) canopy. Furthermore, neonates exhibited a similar pattern. In the case of the cage study, 78% of neonates were found on leaves, with 72% situated in the upper canopy, 22% in the middle, and 6% in the lower canopy. In the field study, 76% of neonates were located on leaves, and 72% of them were detected in the upper canopy, and again, a minor percentage of neonates were discovered in the middle (23%) and lower (5%) canopy. Therefore, significant interactions in tissue selection confined to the middle or lower part of the canopy do not represent moth selection to lay most eggs and neonates cohort of the population located in the upper part of the canopy.

The tendency to lay eggs in the upper canopy and on leaves appears to correlate with the relative abundance of young leaves in the top part of the plant, as we observed more young leaves in the upper part of the canopy during egg scout. However, we did not record this variable. In addition, the surface area of leaves throughout the soybean plant is greater than any other tissue type. Because of this, it is not clear whether females oviposit most often on leaves at the top of the plant because they are the first tissues they encounter, or because they are the most commonly available tissue type, or because of other abiotic or biotic reasons. Furthermore, ovipositional selection could be influenced by the number of leaves on the upper canopy.

Additionally, there are 4 predator species (*Nabis americaferus*, *Nabis rufusculus*, *Geocoris punctipes*, and Phalangiidae) observed predating *H. zea* eggs in soybeans, which are responsible for 13% to 51% of egg predation (Pfannenstiel and Yeargan 2002). In another study, predators (*Nabis* spp., *Geocoris punctipes* (Say), *Orius insidiosus* (Say), Thomisidae) imposed about 70% mortality of *H. zea* eggs that were placed for 24 h in the adaxial part of a soybean leaflet (Anderson and Yeargan 1998). *Helicoverpa zea* might lay more eggs in the abaxial part of the leaves because the predation of their offspring is higher on the adaxial portion. However, there is no data to support a difference in predation in soybeans by leaf side. Furthermore, we did not quantify immature population predation in our study. Therefore, these differences could be unrelated to predation. For example, we also did not quantify canopy biomass. Nonetheless, the upper canopy visually appeared to have more leaves than the middle and lower canopy across all soybean varieties, which might influence the increase in oviposition on that plant part and tissue types.

Our findings are consistent with other studies in related species. For example, studies conducted in Thailand found that *H. armigera* (closely related to *H. zea*) initially tended to lay eggs on “young” and “soft-expanded” cotton leaves and then gradually switched to bracts as the plant matured and had fewer young leaves (Mabbett and Nachapong 1984). In cotton, even though *H. zea* larvae are more common on cotton bolls in the middle and lower section of the cotton plant closer to the main stem; adult females tend to oviposit most eggs in the top third of the canopy on cotton leaves (Braswell et al. 2019a, 2019b). These results are informative to our findings in soybeans. Both *H. armigera* and *H. punctigera* tended to lay eggs on fully expanded leaves at the top of the canopy (Duffield and Chapple 2001). We also found more *H. zea* eggs on the upper part of the canopy on leaves, but we did not record leaf size or node within the top of the canopy.

Our results do not align with all studies. For example, in an apparent mismatch between pest and plant phenology, *H. zea* tends to lay eggs on okra (*Abelmoschus esculentus* L. Moench) leaves during the latter part of the season when the availability of reproductive tissues declines (Latheef and Ortiz 1983). In contrast, we did not observe any ovipositional events later than the R2 stage in our studies. Even though our research only reports egg and neonate sampling at the beginning of the reproductive tissue stage (R1–R2), we continued to sample our plots throughout the season (R1–R6) for another experiment; we found only large larvae, not eggs or small larvae, from beginning pod (R3) to beginning seed (R5). Our study contributes to the extensive body of literature confirming that *H. zea* primarily oviposits on soybean leaves during the flowering stages (Possebom 2023). This behavior is likely an adaptation to facilitate neonate Lepidoptera establishment (Zalucki et al. 2002). Later larval instars can then penetrate large pods to feed on seeds during later developmental stages (McWilliams 1983, Reisig et al. 2021).

Females may be guided by a combination of chemical cues when they make ovipositional choices (Jones et al. 1970); for example, sweet corn silk attracts *H. zea*, and the silk volatiles stimulate them to produce sex pheromones (Raina et al. 1992). In addition, *H. zea* females can detect trichome volatiles and will choose to lay eggs on leaves with more trichomes in wild tomato species (*Lycopersicon hirsutum*) (Juvik et al. 1988). Finally, when *H. armigera* moths are exposed to tobacco flowers, they are attracted to the odors (Cunningham et al. 2004). Nutrition, quantitatively defined as the food consumed, digested, excreted, and converted into biomass that has an effect on insect growth, is an important factor in immature insect development. Furthermore, the nutritional needs of immature insects change as they grow (Scriber and Slansky 1981). Although it has not been experimentally demonstrated, it is plausible that *H. zea* adults choose ovipositional locations based on the nutrition value of plant tissue for their offspring’s development, as specific tissues can fulfill the dietary requirements for insect development and growth, encompassing essential amino acids and vitamins (Hinton 1956). The nutrition provided by the plant tissue could be used for larval feeding after egg hatching. In a greenhouse study, 1-day-old larvae exhibited a tendency for soybean leaves and pods over flowers (Bailey 1979), suggesting that leaves alone could potentially provide the supplements needed for *H. zea* survival. In addition to the leaf tissue itself, larvae will feed on leaf surface structures, such as trichomes (Mueller and Engroff 1980). However, trichomes can be an obstacle for neonates to feed on leaves, as larvae need to first remove the trichomes to access the leaves (Shelomi et al. 2010). Moreover, moths might choose to lay their eggs in the upper canopy leaves independent of nutrition since neonates can spin down in the lower canopy and access tissue throughout the plant (Terry et al. 1989). Neither hypothesis is mutually exclusive. One study showed that *H. zea* could reach their pupal stage when second-instar larvae exclusively consume newly and fully emerged soybean trifoliate. However, they are unable to reach this stage solely through the consumption of flowers, pods, and stems (Suits et al. 2017). Leaves alone could potentially provide the supplements needed for *H. zea* survival, but as larvae grow larger, they shift towards feeding on other plant structures, particularly reproductive tissues such as young pods in the R3 soybean stage (Mueller and Engroff 1980). Furthermore, the nutritional intake of *H. zea* larvae during their later growth stages plays a significant role in affecting the reproductive capacity and lifespan of the insect once they become an adult. This outcome is contingent upon the type of food source on which the larvae developed; for instance, female *H. zea* that were raised on corn kernels laid 4 times as many eggs as those reared on alfalfa foliage (Isely 1935, Lozina-Lozinskii 1939, Hardwick 1965). Corn silk, leaves, and the developing kernels within the corn husk provide essential nutritional components necessary for larval development (Callahan 1956, Dicke and Guthrie 1988, Capinera 2000). Mothers tend to make oviposition choices to maximize their offspring’s survival, increase fecundity, and modify the descendant’s phenotype (Refsnider and Janzen 2010). Consequently, adult moths might choose to lay eggs on soybean leaves, which can provide complete nutrition for offspring survival (Terry et al. 1987a, 1987b, Eckel et al. 1992c), although not all oviposition-site choice hypotheses (Refsnider and Janzen 2010) have been tested for this species.

Plant architecture and leaf structures vary among soybean varieties (Navasero and Ramaswamy 1991) and potentially differ between growth habits. This variation might influence *H. zea* oviposition. For example, determinate varieties typically have fewer nodes and limited vegetative growth, while indeterminate varieties typically have longer stems and a prolonged duration of reproductive tissue availability (Hartung et al. 1981). Also, plant breeders aim to enhance canopy architecture to optimize light distribution and ensure uniform photosynthesis across the canopy to ultimately improve yield (Song et al. 2017). Finally, as mentioned previously, indeterminate varieties have blooms available for an extended period of time, and moths feed on flower nectar (Adjei-Maafo and Wilson 1983a, 1983b). Despite these differences in plant growth patterns, we did not observe a significant difference in ovipositional behavior between growth habits under field or greenhouse conditions. Ovipositional selection can also be influenced by abiotic and biotic factors; for example, *H. zea* might choose to lay its eggs on top of the canopy because temperature is higher at the top of the canopy than in the middle and lower canopy. If the temperature is higher (30–36 °C, Butler 1976), this will decrease the duration between oviposition and hatching (Quaintance and Brues 1905, Ellington and El-Sokkari 1986, Qayyum and Zalucki 1987). Consequently, their offspring could spin down through the canopy to look for other tissues to feed on and would be less exposed to potential predation and parasitism in the upper canopy (assuming predators and parasites target eggs in the upper canopy). Density dependent factors could also be important. For example, leaves may provide a surface to avoid intra-specific cannibalism since they have a greater surface area compared to flowers and pods. Ovipositional selection on leaves, therefore, could reduce opportunities for cannibalism among conspecifics or competition for food.

Our results may have applied implications for IPM. For example, knowing the specific tissue moths tend to lay their eggs in crops such as soybeans may be important if management practices change. This might influence moth behavior due to changes in selection from the management practices. Furthermore, current scouting procedures using the sweep net and beat cloth are focused on *H. zea* larva numbers. Our results could be used to develop scouting procedures or thresholds for eggs, perhaps in combination with numbers of beneficial insects. Or perhaps egg scouting can be used as a way to eliminate fields that may not need to be scouted for larvae at a later date. We know *H. zea* tends to lay more eggs on leaves at the beginning of the reproductive tissue stage (R1–R2). If fields can be identified with eggs under a certain threshold at this time, perhaps these fields do not need to be scouted for this insect for the remainder of the season.

In conclusion, we found that egg and neonate distribution, both vertically and across tissue types, was consistent between growth habits. These results improve our understanding of *H. zea* ecology in soybeans and demonstrate that ovipositional selection is likely independent of reproductive tissue availability between growth habits. Future research should investigate if *H. zea* chooses to oviposit on leaves due to nutritional factors, proportion of the surface area, plant architecture, or other factors.
